# Methyl Esterification Combined with Gas Chromatography-Mass Spectrometry (GC-MS) for Determining the Contents of Lubricant to Evaluate the Compatibility of Chlorinated Butyl Rubber Stoppers with Liposome Injections

**DOI:** 10.1155/2020/9760580

**Published:** 2020-09-01

**Authors:** Xun Gao, Kexin Chen, Miaomiao Chi, Kunming Qin

**Affiliations:** School of Pharmacy, Jiangsu Key Laboratory of Marine Pharmaceutical Compound Screening, Co-Innovation Center of Jiangsu Marine Bio-industry Technology, Jiangsu Ocean University, 59 Cangwu Road Haizhou District, Lianyungang, Jiangsu, China

## Abstract

A simple, sensitive, and exact methyl esterification in combination with gas chromatography-mass spectrometry (GC-MS) method was developed to determine the contents of palmitic acid and stearic acid in the chlorinated butyl rubber stoppers and liposome injections in order to evaluate the compatibility of pharmaceutical packaging materials. In this experiment, palmitic acid and stearic acid were detected in the form of methyl hexadecanoate and methyl stearate in chlorinated butyl rubber stoppers and liposome injections. The results showed good linearities in the range of 0.50–10.00 *µ*g·mL^−1^ for methyl hexadecanoate and 1.00–20.00 *µ*g·mL^−1^ for methyl stearate, with the limits of detection (LOD) 11.94 ng·mL^−1^ and 11.90 ng·mL^−1^, respectively. The recoveries that ranged from 95.25% to 100.29% were satisfied, and the relative standard deviation (RSD) was no more than 7.16%. The developed method was successfully applied to evaluate the compatibility of chlorinated butyl rubber stoppers with liposome injections and the safety assessment.

## 1. Introduction

The quality of the drug was not only related to itself, human's operation, environmental conditions, etc., but also related to the packaging materials, importantly, containing ampoule, vial, cassette bottles, prefilled injection bottles, polyester bottles, infusion bags, or infusion bottles, etc., which were in direct contact with the drug [1]. However, there might occur absorption, adsorption, infusion, permeation, or some chemical reactions between the pharmaceutical packaging materials with the drug, which caused the reduction of active ingredients and the long-term stability of the drug, and even affected the medication safety, seriously [2, 3]. Therefore, it was extremely essential to study the compatibility between pharmaceutical packaging materials and drug for ensuring the quality of the drug by the tests of extraction and migration [4, 5].

As a kind of pharmaceutical packaging material, chlorinated butyl rubber stoppers were widely used in the field of packaging materials due to its considerable advantages, such as inherent cleanliness, biological properties, tightness, and chemical stability [6, 7]. However, owing to the complex compositions of the rubber stoppers and the denseness gradients of the added raw materials, some raw materials, reaction intermediates, decomposition products, vulcanizators, accelerator'promoters, and antioxidants were inevitably left in the internal structure of the rubber stoppers during the process of manufacturing chlorinated butyl rubber stoppers. Under the conditions of storage or sterilization at a higher temperature, these substances could easily migrate to the outer surface of the rubber stoppers, thus affecting the safety and effectiveness of the drug [8, 9]. Consequently, in order to isolate the direct contact between the rubber stoppers and the drug, a strong chemical inert and soft coating might be applied to cover the outer surface of the rubber stoppers to improve the compatibility of them. In this investigation, the chlorinated butyl rubber stoppers covered with Teflon had very low gas permeability, high internal cleanliness, thermal stability, chemical resistance, and good lubricity, which were innoxious, tasteless, unpolluted, and antiaging [10, 11]. However, in the process of manufacturing the kinds of rubber stoppers, fatty acids were frequently used as lubricants [6], which ensured that rubber stoppers could effectively revolve on the conveying track with the liquid medicine during the canning process. Stearic acid will affect the viscosity and vulcanization performance of rubber plugs. If the drug and rubber plug contact, it will affect the quality of drugs [12]. Therefore, it is considered to determine the content of stearic acid in chlorinated butyl rubber stoppers. As we all know, the National Medical Products Administration promulgated the guiding principles for the compatibility Research Technology of Chemical Injections and Plastic Packaging Materials in September 2012, to standardize the design ideas and requirements for the research on the compatibility of injections and plastic packaging materials [13]. Meanwhile, European Pharmacopoeia 8.0 established standards for packaging materials, containers, and the amounts of additives in the compatibility test, which could provide a preliminary basis for determining whether the packaging materials meet the requirements: the content of stearic acid less than 0.5% [14].

Regarding liposome injection, as a special injection, there is no literature to guide whether some components may be changed due to different chemical properties, pH value, storage time, drug excipients, chemical composition, ion state, and so on. These components may leach into pharmaceuticals after long-term storage, which influences the stability and safety of pharmaceutical products. Therefore, the investigation of packaging-drug compatibility is a high priority.

At present, the determination methods of fatty acid content include gas chromatography (GC), gas chromatography-mass spectrometry (GC-MS), liquid chromatography-mass spectrometry ((LC)), and liquid chromatography-mass spectrometry (LC-MS). In addition, there are capillary electrophoresis (CE), near-infrared absorption spectroscopy (NTR), ultraviolet-visible spectrophotometry (UV-VIS), Raman spectroscopy (Ram), nuclear magnetic resonance spectroscopy (NMRS), and so on. Thin layer chromatography is only used in the pretreatment stage of GC and LC, and there are no reports for the separate analysis of fatty acids. At present, GC is the main method for the analysis of fatty acids, which can be used for qualitative and quantitative analysis of most fatty acids, but some isomerized fatty acids can not be completely separated by GC, so it is difficult to determine many kinds of fatty acids at the same time. At present, HPLC is mainly used for the detection of short-chain fatty acids and special fatty acids. LC-MS is often used to determine many kinds of fatty acids at the same time or to study liposome. It is the main method for lipid research. Fourier transform infrared spectroscopy (FTIR) and NMR are new detection methods for the determination of fatty acids in recent years. In recent years, NMR technology has also been applied to the determination of fatty acids. X-ray and Ram are also used in the determination of fatty acids. However, these methods are not compared with GC methods, and their application is not strong. GC-MS has a strong function of structural analysis, and the sample can be analyzed qualitatively without standard [15]. At present, GC-MS is widely used, which is second only to GC for the determination of fatty acids. Advancements in GC have furthered the study of lipids and provided knowledge on fatty acid composition in a short span of time. The economical reasons and availability are always weighed when selecting a detector, and for that reason, a flame ionization detector (FID) may prevail over MS, but GC-MS usually provides lower LOD and limit of quantitation (LOQ) values than GC-FID, and it is able to perform quantitative analyses even below the level of *μ*g/kg [16]. Therefore, GC-MS has higher selectivity, accuracy, and sensitivity than GC-FID.

Esterification, the conversion of fatty acids into methyl esters, is commonly used to analyze fatty acids and to reduce the adsorption of solutes on the support and the surface of the column and improve compound separation [17, 18]. The derivatization methods most commonly used in GC analysis involve the transesterification of acylglycerols and the esterification of free fatty acids into FAME. This process is also called methylation [19, 20]. The reagents most used in acid catalysis esterification are hydrochloric acid (HCl), sulfuric acid (H2SO4), and boron trifluoride (BF3) in methanol [21]. The reaction time of BF3 method is short and the reaction temperature is moderate so that the unsaturated fatty acids will not be destroyed. The advantage of this method is that the methyl esterification of fatty acids is complete, and it is suitable for the methyl esterification of free fatty acids. Thus, BF3 method is more suitable for the analysis of stearic acid and palmitic acid, which is a methyl esterification process in Chinese Pharmacopoeia [22]. However, BF3 is highly toxic and easy to volatilize, and it is easy to turn into harmful substances such as hydrogen fluoride when heated, so its operation and labor protection requirements are higher. In the process of sample treatment, palmitic acid and stearic acid in chlorinated butyl rubber plug were derivatized into volatile methyl ester compounds with methyl palmitate and stearic acid as control, and then GC-MS analysis was carried out, which effectively solved the problem that stearic acid was not easy to be directly analyzed by GC-MS. The analysis of palmitic acid expands the detection range of the method and makes a more comprehensive evaluation of the analytical method.

In this study, the samples containing palmitic acid and stearic acid were operated by the process of methyl esterification in the Chinese Pharmacopoeia and a simple, sensitive, and exact GC-MS method for determining the contents of the target analytes to evaluate the compatibility of chlorinated butyl rubber stoppers and liposome injections was established. In a word, the results of this study would not only provide a theoretical basis for the compatibility between packaging materials and drugs but also a preliminary evaluation for the safety of the liposome injection.

## 2. Experimental

### 2.1. Chemicals and Reagents

Chlorinated butyl rubber stoppers and liposome injections (181102, 181201, and 181203) were purchased from Shenyang Yaoda Yaoyun Pharmaceutical Co., Ltd. (Shenyang, China). The standards of methyl hexadecanoate (99%) and methyl stearate (99%) were bought from Sigma-Aldrich (Shanghai, China) and NU-CHEK PREP.JNC (Tianjin, China), respectively. Methanol and n-heptane of chromatographic grade were acquired from Tianjin Kangkede Technology Co., Ltd. (Tianjin, China) and Shanghai SiXin biotechnology Co., Ltd. (Shanghai, China), respectively. Trifluoroboron of analytical grade was supplied by Bailingwei Technology Co., Ltd. (Beijing, China). Other analytical reagents were provided with Sinopharm Chemical Reagent Co., Ltd. (Shenyang, China), such as potassium hydroxide, sodium chloride, and sodium sulfate.

### 2.2. Preparation of Standard Solution

The stock solutions of methyl hexadecanoate and methyl stearate were prepared in n-heptane to obtain the concentrations of 1.000 mg·mL-1 and 2.000 mg·mL-1, respectively. And the work solutions and calibration curves were prepared from the dilution of stock solutions with n-heptane. All the solutions were stored in volumetric bottles and placed in a refrigerator at 4°C for subsequent experiments.

### 2.3. Preparation of Samples

Accurately 5.0 g of the cracked chlorinated butyl rubber stoppers and 20.0 mL of the potassium hydroxide-methanol (0.50 mol·L-1) solution, placed in the round bottom flask with a volume of 250 mL, firstly were methylated by heating and refluxing for 30 min in a water bath at 65°C. After cooling, 20.0 mL of 15% trifluoride methanol solution as a catalyst, was added at the same heating time and temperature. And then, 40.0 mL of n-heptane was added after the solution cooled, and the reaction was continued for 5 min at the same temperature. Subsequently, 100.0 mL of saturated sodium chloride solution was added to wash the prepared solution after the solution cooled to room temperature. After the solution was shaken thoroughly for a while, the supernatant liquid was removed and washed three times with 20.0 mL of water each time. Finally, the obtained supernatant liquid was dried with anhydrous sodium sulfate and diluted 20 times with n-heptane to gain a sample solution 1 [23].

Meanwhile, 5.0 mL of the liposome injections was accurately weighed and operated according to the above methyl esterification process. And then, the supernatant liquid was diluted 50 times with n-heptane to obtain a sample solution 2.

### 2.4. Instruments and Conditions

**Figure 1:**
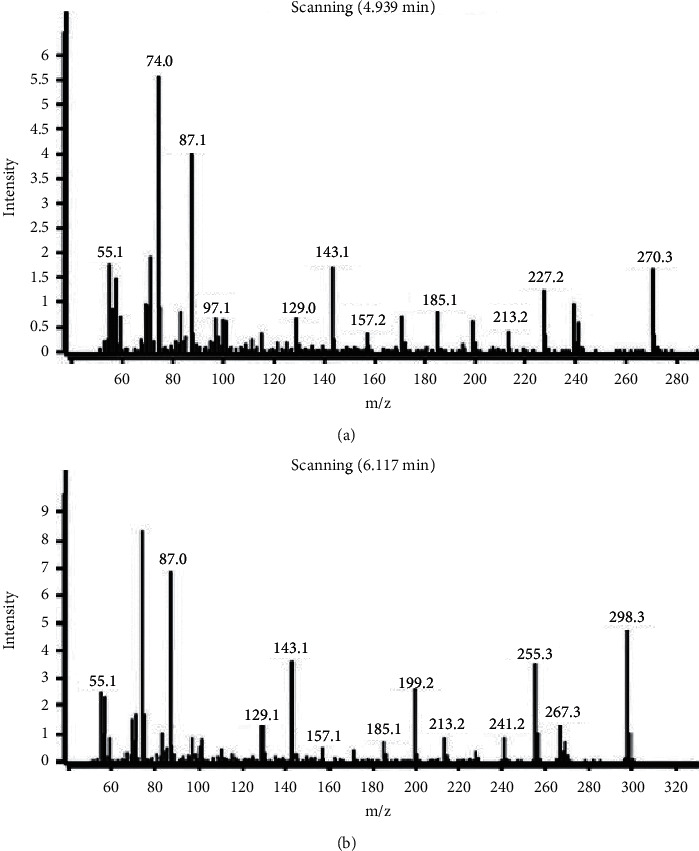
Mass spectrum of methyl hexadecanoate (a) and methyl stearate (b).

This experiment was implemented with an Agilent 6890N-5973 system, which employed an HP-INNOWAX capillary column (30 m × 0.32 mm, 0.25 *µ*m) to separate targets. Helium was used as the carrier gas at a flow rate of 1.0 mL·min^−1^. The initial oven temperature was kept at 150°C for 3 min, then raised to 210°C at a rate of 50°C·min^−1^, and kept for 4 min. The temperature of the electron impact ionization (EI) and inlet was 230°C, 200°C, respectively. The injection volume was 1 *µ*L at splitless mode. Selective ion-monitoring (SIM) mode was adopted and the optimal ion of the two target analytes was 74.0, whose mass spectra were shown in Figure 1. Moreover, the solvent delay time was set at 2.5 min to protect the filament.

### 2.5. Method Validation

This experiment would verify the reliability of the method from the aspects of specificity, sensitivity, linearity, precision, repeatability, accuracy, and stability. The specificity of the method was assessed by contrasting the chromatograms of blank reagent (n-heptane), standard solutions, and sample solutions. It was the blank reagent and the sample solutions that should have no interference with each other. Meanwhile, the resolution of the two target analytes in the mixed standard solution was greater than 1.5. The lowest analytical amounts of analytes were expressed by the concentration of LOD and LOQ, and the corresponding signal-to-noise (S/N) was 3 and 10. The linearity of the method was investigated in the concentration range of 0.50–10.00 *µ*g mL^−1^ for methyl hexadecanoate and 1.00–20.00 *µ*g mL^−1^ for methyl stearate. The precision of the instrument was determined by measuring the standard solutions of methyl hexadecanoate (1.00 *µ*g mL^−1^) and methyl stearate (2.00 *µ*g mL^−1^) for six times. Repeatability was evaluated by the determination of RSD of target analytes in the samples. To verify the recovery of method, samples were spiked at three concentration levels to compare the contents of the target analytes. In this study, due to the mickle contents of the two analytical targets in the chlorinated butyl rubber stoppers and liposome injections, the amounts of standard substances were greatly increased to make the measurement more precise. In order to evaluate the stability, the sample solutions were placed at room temperature for 0, 6, and 12 h, and measured at the time point, respectively. At last, the results of the stability were assessed by the RSDs of the peak areas.

### 2.6. Extraction and Migration Tests

The chlorinated butyl rubber stoppers were treated by methyl esterification to detect whether the target analytes including methyl hexadecanoate and methyl stearate were contained. Referring to the recommendations of the International Conference on Harmonization of Technical Requirements for Registration of Pharmaceuticals for Human Use (ICH) guidelines [24], the liposome injections used with the chlorinated butyl rubber stoppers were stored under the accelerated aging conditions (25°C ± 2°C, relative humidity 60% ± 10%) for 3 months to detect the amounts of methyl hexadecanoate and methyl stearate in the chlorinated butyl rubber stoppers into the liposome injections and investigate the compatibility of them [23].

## 3. Results

### 3.1. Method Validation

#### 3.1.1. Specificity

**Figure 2:**
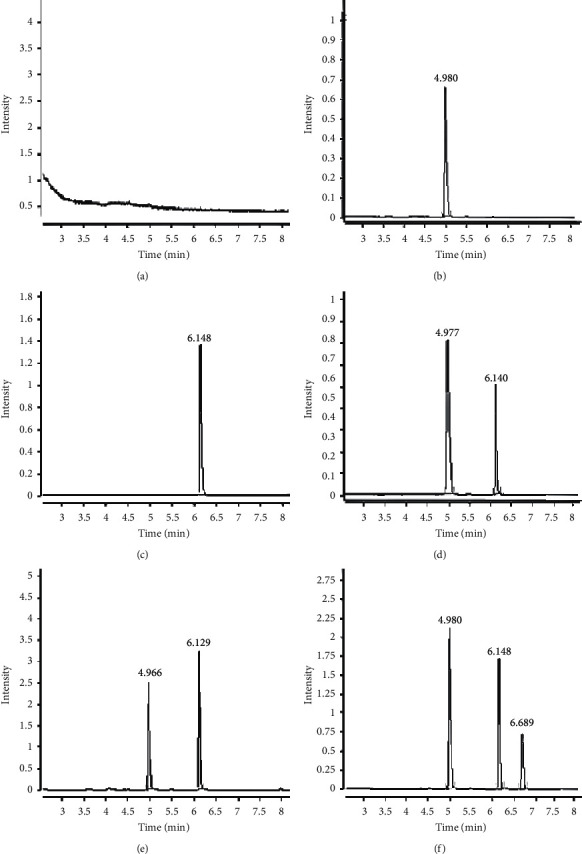
Chromatogram of blank reagent (a), methyl hexadecanoate solution (b), methyl stearate solution (c), mixed standard solution (d), sample solution 1 (e), sample solution 2 (f).

As shown in Figure 2, the retention times of methyl hexadecanoate and methyl stearate were 4.977 min and 6.140 min, respectively. The blank reagent and the sample solution did not interfere with each other, and the resolution of target analytes in the mixed standard solution was higher than 1.5, which indicated that the method had good specificity.

#### 3.1.2. LOD and LOQ

The LOD of the target analytes in the mixed standard solution were 11.94 ng·mL-1 and 11.90 ng·mL-1 with the S/N of 3, respectively. According to the S/N of 10, the LOQ xwas 39.80 ng·mL-1 and 39.68 ng·mL-1, respectively. The results showed that the sensitivity of the method was satisfactory.

#### 3.1.3. Linearity

With the linear regression analysis, the calibration curves were acquired with the concentration range of 0.50–10.00 *µ*g mL^−1^ for methyl hexadecanoate and 1.00–20.00 *µ*g mL^−1^ for methyl stearate. The equations of calibration curves were *y* = 664611 *x* − 14659 (*R*^2^ = 1) and *y* = 147905 *x* − 13003 (*R*^2^ = 0.9998), respectively, which demonstrated the linear relationship was excellent.

#### 3.1.4. Precision and Repeatability

The precision of the instrument was determined by injecting the standard solution for 6 times. The results showed the RSDs of the retention time was less than 0.03% and the peak areas less than 1.30%, which indicated that the instrument had good precision.

The repeatability of the method was measured by analyzing samples for 6 times and the RSDs of the two target analytes were no more than 2.21%, indicating that the method had satisfactory repeatability.

#### 3.1.5. Accuracy

The accuracy of this method was determined by spiking analytes free matrix in 3 replicates at three different concentration levels (low, medium, high) followed by extraction. As shown in Tables 1 and 2, the experimental results showed that the average recoveries were from 95.25% to 100.29% with RSDs less than 7.16%, revealing that the accuracy was applicable.

#### 3.1.6. Stability

The processed sample solutions were placed at room temperature for 0, 6, and 12 h in order to investigate the stability studies. The results showed the RSDs less than 4.73% for the peak area of the two target analytes, which demonstrated that the sample solutions were stable within 12 h and the proposed method had good stability.

### 3.2. Extraction Tests

In the extraction tests, the contents of methyl hexadecanoate and methyl stearate in the chlorinated butyl rubber stoppers were determined by the detection of two sample solutions prepared in parallel. From Table 3, the average contents of methyl hexadecanoate (*M* = 270.00) and methyl stearate (*M* = 298.51) in the sample solutions were 0.4774 mg g^−1^ and 0.6596 mg g^−1^, respectively. According to the reduction formula by Xie [6], the average contents of palmitic acid (*M* = 256.42) and stearic acid (*M* = 284.48) in the sample solutions were 0.4534 mg g^−1^ and 0.6286 mg g^−1^, respectively.

### 3.3. Migration Tests

In order to evaluate the compatibility of packaging materials and liquid, the samples were required to be stored under certain conditions and then the contents of the target analytes were detected whether it migrated from chlorinated butyl rubber stoppers to liposome injections. In this test, the accelerated aging conditions (25°C ± 2°C, relative humidity 60% ± 10%) were applied at 0 and 3 months, and the contents of target analytes in the liposome injections were shown in Table 4.

## 4. Discussion

### 4.1. Source of Fatty Acids in the Samples

The reason why palmitic acid and stearic acid were detected in the extraction tests was that they were added as lubricants during the preparation of the chlorinated butyl rubber stoppers. On the other hand, some pharmaceutical excipients were added constantly in the pharmaceutical manufacturing process, such as Tween 40 (Polyoxyethylene sorbitan monopalmitate), Tween 60 (Polyoxyethylene sorbitan monostearate), and so on, in order to improve the properties of the drug and enhance the stability or increase the solubility of the drug [25]. After the hydrolysis of Tween 40 and Tween 60, palmitic acid and stearic acid were found. Therefore, palmitic acid and stearic acid in the liposome injections might be derived from the hydrolysis of pharmaceutical excipients.

### 4.2. The Selection of Injection Mode

The mode of injection was divided into two types, split injection and splitless injection. Compared with split injection, splitless injection had significantly higher sensitivity and was commonly used for the analysis of environment, clinic, and pharmacy. But the pretreatment of the splitless injection samples must be paid more attention to protect the column from contamination. At the same time, the splitless injection had stricter requirements on the sample solvents. In general, the use of high boiling solvents was advantageous over low boiling solvents, and the polarity of the solvents must match the polarity of the samples, and that the solvent peaks were tested before all the components of the samples to prevent the early outflow peaks from being masked by the large peak of the solvents. For the split injection, most of the samples were vented, and only a small portion of the samples entered the column, which largely prevented solid particles and nonvolatile sample components from entering the column and avoided the problem of column contamination. More importantly, this mode could effectively avoid the remaining solvents, which would dilute the samples and cause the poor reproducibility. Due to the high degree of clarification of the two sample solutions, a splitless mode was used in this experiment, but on account of the high contents of the two target analytes in the samples, it was best to use a split injection mode [26, 27].

### 4.3. The Cause of Methyl Esterification

With a high boiling point, the fatty acid requires high temperature condition, which would cause a certain loss in the analysis process and damage in the capillary column. To reduce the boiling point and improve the stability, fatty acid methyl ester should be formed by the process of methyl esterification [22]. However, during the analysis, the contents of ester were different due to the less difference in the time and temperature of methyl esterification, so the same temperature and time should be controlled as much as possible in the experiment.

### 4.4. Safety Assessment

Although the results showed that fatty acids did not migrate from pharmaceutical packaging materials to liposome injections, the potential migrants from chlorinated butyl rubber stoppers were predicted with the use of LOD to evaluate the potential risk. The LODs of methyl hexadecanoate and methyl stearate were 11.94 ng mL^−1^ and 11.90 ng mL^−1^, respectively. On the basis of a report by the International Uniform Chemical Information Database, the no-observed-effect levels (NOEL) of target analytes were 5000 mg·kg^−1^ day^−1^ (rat, oral, 150 days) and 5000 mg·kg^−1^ day^−1^ (rabbit, oral, short-term), respectively [28]–30].

With the clinical data, the maximum daily dose of liposome injection is 500 mg. From the safety point of view, the potential maximum daily intake of palmitic acid and stearic acid was 1.19 mg, respectively, estimated by the LODs.

## 5. Conclusion

The results of the compatibility test between the rubber plug and the drug can provide information about whether the rubber plug causes the migration, adsorption, and even chemical reaction of the principal components of the drug, makes the drug invalid, and even produces serious toxic and side effects, so as to ensure the safety, effectiveness, and uniformity of the drug. The compatibility test between drug packaging materials and drugs is the most intuitive and effective method to evaluate the performance and quality of drug packaging materials. A simple, effective, and precise method based on GC-MS to detect the possible migrates in chlorinated butyl rubber stoppers was developed, which was successfully used to study the compatibility of chlorinated butyl rubber stoppers with liposome injections and the safety assessment. The results showed that fatty acids did not migrate from pharmaceutical packaging materials to liposome injections, indicating a safety medication for patients. Accurate and unbiased results provided a theory for the long-term safe storage of liposome injections. This method can be further applied to drugs with different chemical properties, available conditions as pH, storage time, pharmaceutical excipients, the chemical composition of the liposome, and so on.

## Figures and Tables

**Table 1:** Recoveries of methyl hexadecanoate and methyl stearate in chlorinated butyl rubber stoppers.

Analyte	No.	Spiked (mg)	Sample (mg)	Detected (mg)	Founded (mg)	Recovery (%)	Average recovery (%)	RSD (%)
Methyl hexadecanoate	1-1	0.7960	2.0481	2.7907	0.7426	93.29	97.33	2.55
	1-2	0.7936	2.0482	2.8083	0.7601	95.78		
	1-3	0.7975	2.0483	2.8039	0.7556	94.74		
	2-1	0.9950	2.0480	3.0122	0.9642	96.90		
	2-2	0.9920	2.0480	3.0159	0.9679	97.57		
	2-3	0.9969	2.0481	3.0313	0.9832	98.63		
	3-1	1.1939	2.0478	3.2462	1.1985	100.38		
	3-2	1.1904	2.0484	3.2131	1.1647	97.84		
	3-3	1.1963	2.0482	3.2547	1.2065	100.85		
Methyl stearate	1-1	1.4285	2.6063	3.9752	1.3689	95.83	96.55	1.29
	1-2	1.4370	2.6064	4.0012	1.3949	97.07		
	1-3	1.4299	2.6065	3.9971	1.3906	97.25		
	2-1	1.7856	2.6061	4.3496	1.7435	97.64		
	2-2	1.7963	2.6061	4.3094	1.7033	94.82		
	2-3	1.7874	2.6062	4.3377	1.7315	96.88		
	3-1	2.1427	2.6058	4.6887	2.0829	97.21		
	3-2	2.1555	2.6066	4.6410	2.0344	94.38		
	3-3	2.1448	2.6063	4.7059	2.0996	97.89		

**Table 2:** Recoveries of methyl hexadecanoate and methyl stearate in liposome injections.

Analyte	No.	Spiked (mg)	Sample (mg)	Detected (mg)	Founded (mg)	Recovery (%)	Average recovery (%)	RSD (%)
Methyl hexadecanoate	1-1	5.5717	1.3377	7.0856	5.7478	103.16	95.25	7.16
	1-2	5.5551	1.3377	7.0581	5.7204	102.98		
	1-3	5.5828	1.3377	7.1345	5.7967	103.83		
	2-1	6.9647	1.3377	7.9494	6.6117	94.93		
	2-2	6.9439	1.3377	7.8850	6.5473	94.29		
	2-3	6.9785	1.3377	7.9809	6.6432	95.19		
	3-1	8.3576	1.3377	8.6645	7.3268	87.67		
	3-2	8.3326	1.3377	8.6407	7.3030	87.64		
	3-3	8.3742	1.3377	8.6670	7.3293	87.52		
Methyl stearate	1-1	6.3487	0.9936	7.4200	6.4263	101.22	100.29	1.07
	1-2	6.3867	0.9936	7.4009	6.4073	100.32		
	1-3	6.3550	0.9936	7.4757	6.4821	102.00		
	2-1	7.9358	0.9936	8.9891	7.9954	100.75		
	2-2	7.9834	0.9936	8.9131	7.9195	99.20		
	2-3	7.9438	0.9936	9.0308	8.0371	101.18		
	3-1	9.5230	0.9936	10.4704	9.4768	99.51		
	3-2	9.5800	0.9936	10.4637	9.4701	98.85		
	3-3	9.5325	0.9936	10.4876	9.4940	99.60		

**Table 3:** Contents of target analytes in chlorobutyl rubber stoppers.

No.	Methyl hexadecanoate (mg·g^−1^)	Palmitic acid (mg·g^−1^)	Methyl stearate (mg·g^−1^)	Stearic acid (mg·g^−1^)
1	0.4751	0.4512	0.6563	0.6255
2	0.4796	0.4555	0.6628	0.6316
Average contents	0.4774	0.4534	0.6596	0.6286

**Table 4:** Contents of target analytes in liposome injections.

Conditions	Batch NO.	Methyl hexadecanoate (mg^.^mL^−1^)	Ester contents (mg^.^mL^−1^)	Acid contents (mg^.^mL^−1^)	Methyl stearate (mg^.^mL^−1^)	Ester contents (mg^.^mL^−1^)	Acid contents (mg^.^mL^−1^)
0 h	181102-1	1.2028	1.2019	1.1414	0.9595	0.9641	0.9188
	181102-2	1.2010			0.9688		
	181201-1	1.2059	1.1971	1.1369	0.9752	0.9679	0.9224
	181201-2	1.1882			0.9606		
	181203-1	1.1944	1.2056	1.1450	0.9652	0.9763	0.9304
	181203-2	1.2169			0.9873		
3 months	181102-1	1.2011	1.2011	1.1407	0.9581	0.962	0.9168
	181102-2	1.2010			0.9659		
	181201-1	1.2014	1.1996	1.1393	0.9723	0.9664	0.9210
	181201-2	1.1978			0.9605		
	181203-1	1.1998	1.2034	1.1429	0.9669	0.9762	0.9303
	181203-2	1.2070			0.9854		

## Data Availability

The data used to support the findings of this study are available from the corresponding author upon request.
